# Assessing disability-adjusted life years (DALY) from multiple medication use and multiple illnesses of older adults in rural Thailand

**DOI:** 10.1007/s40520-026-03435-y

**Published:** 2026-06-23

**Authors:** Warinmad Kedthongma, Suajin Kasawong, Nitikorn Phoosuwan, Wuttiphong Phakdeekul

**Affiliations:** 1 Faculty of Public Health, Kasetsart University, Chalermphrakiat Sakon Nakhon Province Campus, Sakon Nakhon Province, Thailand; 2 Phon Sawan Hospital, Phon Sawan District, Nakhon Phanom Province, Thailand; 3 Faculty of Public Health, Thammasat University, Pathum Thani, Thailand; 4 Department of Public Health and Caring Sciences, Faculty of Medicine, Uppsala University, Uppsala , Sweden

**Keywords:** Disability-adjusted life years, DALYs, Polypharmacy, Multimorbidity, Potentially inappropriate medications, Older adults, Rural Thailand, Disease burden

## Abstract

**Background:**

Geriatric syndromes including polypharmacy and multimorbidity represent growing public health challenges in aging rural populations, yet their population-level burden remains poorly quantified in low- and middle-income countries.

**Objective:**

This study aimed to quantify gross disease burden, expressed as disability-adjusted life years (DALYs), across 25 conditions in a large rural Thai older adult population. Particular emphasis was placed on polypharmacy, multimorbidity, and potentially inappropriate medications, alongside the identification of disease clustering patterns to inform integrated care planning.

**Methods:**

Population-based cross-sectional study of 587,905 older adults (aged ≥ 60 years) from 46 secondary hospitals in the 8th Health Service Region of northeastern Thailand. DALYs were calculated using Global Burden of Disease 2017 methodology. Polypharmacy was defined as concurrent use of ≥ 5 unique active pharmaceutical ingredients (APIs) for ≥ 90 consecutive days, assessed over both 2-year and 3-year lookback windows. Multimorbidity was defined as ≥ 2 chronic conditions. Potentially inappropriate medications were assessed using 2019 American Geriatrics Society Beers Criteria and Thai Rational Drug Use (RDU) criteria. DALY estimates represent gross population-level burden attributable to each condition or syndrome, without adjustment for co-occurring conditions.

**Results:**

Total disease burden was 48.4 million DALYs (8,240,612 DALYs per 100,000 population). Diabetes mellitus accounted for the highest burden (16,511,859 DALYs; 2,910,112 per 100,000), followed by hypertension (8,331,200 DALYs; 1,417,720 per 100,000). Polypharmacy affected 59.5% (*n* = 349,803) contributing 4,035,612 DALYs (686,408 per 100,000), with YLL comprising 54.3% of this burden. Multimorbidity affected 48.6% (*n* = 285,721) contributing 3,301,572 DALYs. Potentially inappropriate medications were present in 32.1% (*n* = 188,718). The most common disease clusters were diabetes, hypertension, renal failure and respiratorycardiovascular combinations.

**Conclusions:**

Polypharmacy and multimorbidity represent substantial gross population-level burdens in rural Thai older adults. These findings apply specifically to older adults accessing outpatient services at secondary hospitals in the 8th Health Service Region and should not be generalized to the entire rural older adult population. High prevalence of potentially inappropriate medications indicates urgent need for medication optimization interventions. Diabetes emerges as the leading disease burden despite lower prevalence than hypertension, highlighting the importance of DALY-based priority setting. These gross DALY estimates should be interpreted as descriptive measures of population health loss associated with each condition, rather than causal or adjusted effect estimates.

**Supplementary Information:**

The online version contains supplementary material available at https://doi.org/10.1007/s40520-026-03435-y.

## Introduction

### Population aging and health challenges in rural Thailand

Thailand is experiencing rapid demographic transition, with the proportion of older adults (≥ 60 years) increasing from 10% in 2005 to over 20% in 2022 [1]. Rural areas face disproportionate aging, with older adult populations exceeding 25% in northeastern provinces [2, 3]. This demographic shift coincides with epidemiological transition characterized by rising chronic disease burden, multimorbidity, and complex medication regimens [4, 5]. Rural older adults face substantial healthcare access barriers including limited specialist availability, distance to facilities, and reduced pharmaceutical services compared to urban populations [6–8].

### Disability-adjusted life years as population health metric

Disability-adjusted life years (DALYs) have emerged as the cornerstone metric for quantifying population health loss worldwide [9, 10]. By combining mortality (years of life lost, YLL) and morbidity (years lived with disability, YLD), DALYs enable systematic comparison across diseases, populations, and time periods [11]. The Global Burden of Disease (GBD) study has employed DALYs since 1990 to guide international health policy and resource allocation [10, 12]. However, DALY-based burden assessment in rural older adult populations of low- and middle-income countries remains limited, particularly for geriatric-specific syndromes [13, 14].

### Rationale for applying DALYs to geriatric syndromes

Polypharmacy and multimorbidity are increasingly recognized as major contributors to population health loss beyond their role as correlates of individual diseases. Polypharmacy, typically defined as concurrent use of five or more medications, affects 30–90% of older adult populations globally [15, 16] and has been independently associated with adverse outcomes including falls, hospitalization, and premature mortality, even after adjusting for underlying disease burden [17, 18]. Similarly, multimorbidity the coexistence of multiple chronic conditions affects 23–98% of individuals over 65 years, and imposes cumulative care burdens that exceed the simple sum of individual conditions [19–21].

Existing global burden of disease studies have provided DALY estimates for specific conditions such as diabetes, hypertension, and COPD. The prevalence of polypharmacy and multimorbidity has been extensively documented within these frameworks. Standard prevalence-based analyses can identify the number of individuals affected by geriatric syndromes, yet they fall short of quantifying the associated population-level health loss. This methodological limitation underscores the need for burden-of-disease metrics, such as DALYs, to capture the true magnitude of health loss attributable to these conditions. Polypharmacy and multimorbidity are typically treated as clinical states or care-complexity indicators, not as independent DALY categories in burden of disease frameworks [22–24].

This study is the first to apply the GBD DALY framework to quantify population-level burden of polypharmacy and multimorbidity as distinct entities in a large rural Thai older adult cohort. This enables direct comparison with traditional disease categories, offering a quantitative basis for healthcare priority setting beyond what prevalence data can provide. The reported DALY estimates reflect gross, unadjusted population-level health loss without controlling for co-occurring conditions. They should therefore be interpreted as descriptive burden indicators, not as causal or independent effect estimates.

### Study objectives

This study aimed to quantify gross disease burden using DALYs across 25 conditions in a large rural Thai older adult population (*N* = 587,905) who accessed outpatient services at secondary hospitals in the 8th Health Service Region. Findings therefore pertain to this healthcare-accessing cohort and may not be generalized to the entire rural older adult population in the region, particularly those who do not access formal healthcare services. The four specific aims (1) identifying leading contributors to total disease burden, (2) quantifying polypharmacy and multimorbidity prevalence and associated DALY burden, (3) assessing PIM use, and (4) examining disease clustering patterns collectively address the magnitude and distribution of population health loss attributable to both traditional disease categories and geriatric syndromes in a rural low- and middle-income country context, thereby informing integrated care planning and resource allocation for aging populations.

## Methods

### Study design and population

We conducted a cross-sectional retrospective study using electronic health records from January 2019 to December 2024. The study population comprised older adults aged ≥ 60 years receiving outpatient care at secondary hospitals in the 8th Health Service Region of northeastern Thailand. This is an explicitly healthcare-accessing cohort: the dataset represents individuals who attended outpatient services at the 46 participating secondary hospitals during the study period. Community-dwelling older adults who did not access these services are not represented, and burden estimates should be interpreted accordingly. This region encompasses Nakhon Phanom Province and adjacent provinces, with 46 secondary hospitals selected via random cluster sampling from the 308 secondary hospitals across 20 northeastern provinces. The focus on secondary hospitals reflects the primary care-to-specialist referral pathway in Thailand’s district hospital system. We acknowledge that this approach may underestimate the burden attributable to patients managed exclusively at tertiary centers; this is explicitly noted as a limitation. All hospitals provided de-identified electronic data from the Health Data Center (HDC) in accordance with the Ministry of Public Health standard structure (DAHS_MoPH version 2.4). Eligible patients had ≥ 1 of 22 non-communicable diseases and ≥ 1 prescribed medication. We excluded patients lacking medication information or using exclusively injectable medications. The final study population comprised 587,905 individuals from a database linking hospital encounters, primary care diagnoses, and pharmaceutical dispensing records. Repeated encounters for the same patient were aggregated at the individual level using unique patient identifiers, ensuring that each individual contributed only one observation to the analysis regardless of the number of healthcare visits.

### Disease classification and definitions

We analyzed 25 disease categories representing major contributors to population health loss in rural Thailand [25]. Categories included: cardiovascular diseases (hypertension, ischemic heart disease, cerebrovascular disease, chronic heart disease, stroke, peripheral vascular disease); metabolic conditions (diabetes mellitus, obesity, anemia); respiratory diseases (COPD/emphysema, asthma); renal failure (defined as CKD stage 3b–5, identified by ICD-10 codes N18.3–N18.5 and estimated GFR < 45 mL/min/1.73 m² where available from linked laboratory data) [26, 27]; cancer/neoplasms; mental health conditions (mood disorders); infectious diseases (tuberculosis, HIV); substance-related conditions; chronic heart failure; and geriatric syndromes (polypharmacy, multimorbidity, PIMs) [28–30].

**Polypharmacy** was defined as concurrent use of ≥ 5 unique active pharmaceutical ingredients (APIs) for ≥ 90 consecutive days. The 90-day minimum duration threshold was selected to reflect a clinically meaningful period of sustained concurrent medication use, consistent with published operational definitions of chronic polypharmacy [26, 27], and to distinguish polypharmacy from transient multi-drug prescribing during acute illness episodes. To avoid overestimation due to brand-name switching or generic substitution, medications were classified at the API level using the Anatomical Therapeutic Chemical (ATC) classification system; different brand names or generic formulations of the same API were counted as a single medication. This approach was assessed over both a 2-year and a 3-year lookback window [31, 32].

**Multimorbidity** was defined as coexistence of ≥ 2 chronic conditions from a validated 40-condition list, assessed over a 2-year lookback period. It is important to note that individuals may simultaneously meet criteria for multiple syndromes (e.g., both polypharmacy and multimorbidity), and each syndrome was counted independently in all prevalence and DALY estimates.

**Potentially inappropriate medications (PIMs)** were assessed using the 2019 American Geriatrics Society Beers Criteria and Thai-specific RDU criteria [33, 34].

### DALY calculation

DALYs were calculated following Global Burden of Disease 2017 methodology [10]: DALY = YLL + YLD. Years of life lost (YLL) employed the GBD 2017 reference life table with life expectancy at birth of 86.6 years for females and 80.3 for males. YLL = N × L, where N = number of deaths and L = standard life expectancy at age of death. Complete mortality records were obtained from national civil registration linked to the healthcare database. Years lived with disability (YLD) were calculated as: YLD = P × DW × D. For geriatric syndromes (polypharmacy and multimorbidity), we adopted a conservative proxy approach applying the disability weight of the most prevalent associated chronic condition (diabetes mellitus; DW = 0.049). Consequently, DALYs for different syndromes and conditions should not be summed, as this would result in double-counting. All DALY estimates are gross, descriptive measures of population health loss associated with each syndrome, not estimates of their independent or causal contributions after adjustment for co-occurring conditions [35, 36]. DALY rates were expressed both as absolute numbers and as rates per 100,000 population to facilitate comparison with other studies [37].

### Missing data

Missing data were examined across all variables. Completeness was high: diagnostic codes were available for 98.7% of records, medication data for 97.2%, and mortality linkage for 96.8%. Records with missing medication information or exclusively injectable medications were excluded per the eligibility criteria. No multiple imputation was applied; the primary analysis used complete cases. Sensitivity analyses excluding records with any missing covariate data produced results consistent with the main findings.

### Statistical analysis

Descriptive statistics characterized disease prevalence and DALY burden. Disease clustering patterns were examined using market basket association analysis (Apriori algorithm) implemented in Python 3.8 (mlxtend library), with minimum support of 10% and minimum confidence of 60%. These threshold values were chosen based on standard practice in comorbidity association analysis for large administrative datasets, as they balance pattern interpretability with clinical meaningfulness. Sensitivity analyses using alternative thresholds (support ≥ 8%; confidence ≥ 55%) produced qualitatively similar clustering patterns, supporting the robustness of the five identified clusters. This method identifies gross co-occurrence patterns and does not imply causal directionality. All analyses used Python 3.8 with pandas, numpy, and scipy libraries. Statistical significance was set at *p* < 0.05 [37, 38].

### Ethical considerations

This research was approved by the Human Research Ethics Committee, Provincial Public Health Office, Nakhon Phanom, on October 3, 2023 (approval number REC 056/66). The study utilized de-identified administrative health data in compliance with the Thai Personal Data Protection Act B.E. 2562 (2019). No individual patient consent was required for analysis of de-identified administrative data [39, 40].

## Results

### Study population characteristics

Table 1 presents the baseline characteristics of the 587,905 older adults included in the study. The mean age was 71.3 years (SD 8.6); 53.2% were female. The mean number of chronic conditions was 3.2 (SD 1.8) and the mean number of medications was 5.8 (SD 3.1). Data were drawn from 46 secondary hospitals across the 8th Health Service Region. The dataset represents older adults who accessed outpatient healthcare services during the study period; community-dwelling individuals who did not seek care are not represented.

**Table 1:** baseline characteristics of the study population (*N* = 587,905)

Characteristic	Total (*N* = 587,905)	With Polypharmacy (*n* = 349,803)	Without Polypharmacy (*n* = 238,102)
Age, years, mean (SD)	71.3 (8.6)	72.1 (8.4)	70.1 (8.9)
Age group, n (%)			
60–69 years	241,041 (41.0%)	131,276 (37.5%)	109,765 (46.1%)
70–79 years	210,323 (35.8%)	127,302 (36.4%)	83,021 (34.9%)
≥80 years	136,541 (23.2%)	91,225 (26.1%)	45,316 (19.0%)
Sex, female, n (%)	312,581 (53.2%)	183,211 (52.4%)	129,370 (54.3%)
No. of chronic conditions, mean (SD)	3.2 (1.8)	4.1 (1.7)	1.9 (1.4)
No. of medications, mean (SD)	5.8 (3.1)	7.9 (2.8)	3.0 (1.6)
Selected chronic conditions, n (%)			
Diabetes mellitus	236,337 (40.2%)	172,193 (49.2%)	64,144 (26.9%)
Hypertension	347,451 (59.1%)	231,412 (66.2%)	116,039 (48.7%)
Renal failure (CKD stage 3b–5)	226,931 (38.6%)	163,218 (46.7%)	63,713 (26.8%)
Emphysema/COPD	27,631 (4.7%)	18,224 (5.2%)	9,407 (3.9%)
Cerebrovascular disease	24,692 (4.2%)	16,341 (4.7%)	8,351 (3.5%)
Mood disorders	17,049 (2.9%)	11,892 (3.4%)	5,157 (2.2%)
Ischemic heart disease	14,223 (2.4%)	10,132 (2.9%)	4,091 (1.7%)
Asthma	12,891 (2.2%)	8,643 (2.5%)	4,248 (1.8%)
Cancer/neoplasms	9,642 (1.6%)	7,118 (2.0%)	2,524 (1.1%)
Multimorbidity (≥ 2 conditions), n (%)	285,721 (48.6%)	220,311 (63.0%)	65,410 (27.5%)
PIM (Beers 2019), n (%)	188,717 (32.1%)	141,219 (40.4%)	47,498 (19.9%)
Participating hospitals	46 secondary hospitals, 8th Health Service Region	—	—

SD = standard deviation; CKD = chronic kidney disease; PIM = potentially inappropriate medications. Values from 2-year lookback unless stated. CKD staging corrected to stage 3b–5 to reflect eGFR < 45 mL/min/1.73 m² threshold applied

### Overall disease burden profile

The total gross disease burden across 587,905 individuals was 48,381,551 DALYs (8,240,612 per 100,000 population). Table 2 presents the top-10 conditions by DALY burden, with both absolute and rate-based figures. All DALY estimates represent gross burden (unadjusted for co-occurring conditions). Diabetes mellitus accounted for the largest burden (16,511,859 DALYs; 2,910,112 per 100,000; 34.1% of total) despite 40.2% prevalence, reflecting a high per-person impact driven predominantly by YLD (85.8% of diabetes DALYs). Hypertension ranked second (8,331,200 DALYs; 1,417,720 per 100,000; 17.2% of total). Polypharmacy (2-year window) represented the third-largest burden (4,035,612 DALYs; 686,408 per 100,000), affecting 59.5% of the population. Renal failure contributed 3,781,356 DALYs (643,136 per 100,000), reflecting the diabetes–hypertension–nephropathy axis in this population. Multimorbidity (2-year window) accounted for 3,301,572 DALYs (561,565 per 100,000). Supplementary Table S1 lists all 25 analyzed conditions with full DALY breakdown, including conditions with lower prevalence such as stroke (218,337 DALYs), ischemic heart disease (526,655 DALYs), and tuberculosis (71,559 DALYs).

**Table 2:** Top 10 conditions by DALY burden in rural thai older adult population (*N* = 587,905)

Rank	Condition	Prevalence *N* (%)	YLL	YLD	Total DALYs	DALY rate per 100,000
1	Diabetes mellitus	236,337 (40.2%)	2,342,107	14,169,751	16,511,859	2,910,112
2	Hypertension	347,451 (59.1%)	2,192,421	6,138,779	8,331,200	1,417,720
3	Polypharmacy (2-year window) ★	349,803 (59.5%)	2,193,267	1,842,344	4,035,612	686,408
4	Renal failure (CKD stage 3b–5) ★†	226,931 (38.6%)	1,990,187	1,791,168	3,781,356	643,136
5	Polypharmacy (3-year window) ★	279,842 (47.6%)	1,855,357	1,558,500	3,413,858	580,619
6	Multimorbidity (2-year window) ★	285,721 (48.6%)	1,794,333	1,507,239	3,301,572	561,565
7	PIM Beers 2019 (2-year window) ★	188,717 (32.1%)	1,221,002	1,025,641	2,246,644	382,118
8	Emphysema/COPD	27,631 (4.7%)	226,302	814,688	1,040,990	177,078
9	Cerebrovascular diseases	24,692 (4.2%)	149,633	538,680	688,314	117,082
10	Mood disorders	17,049 (2.9%)	144,748	455,956	600,704	102,175

YLL = Years of Life Lost; YLD = Years Lived with Disability; DALYs = Disability-Adjusted Life Years. DALY rates expressed per 100,000 population. ★ = geriatric syndrome / lookback-window estimates. †CKD staging corrected to stage 3b–5 to reflect eGFR < 45 mL/min/1.73 m² threshold. All estimates are gross (unadjusted)

### Geriatric syndromes: polypharmacy and multimorbidity

Polypharmacy (2-year window) affected 349,803 individuals (59.5%) with a gross burden of 4,035,612 DALYs (686,408 per 100,000). Notably, YLL comprised 54.3% of polypharmacy DALYs, suggesting a disproportionate association with premature mortality beyond disability alone. The 3-year window assessment identified 279,843 individuals (47.6%) with polypharmacy, contributing 3,413,858 DALYs (580,619 per 100,000). The consistency of YLL proportion (54.3–54.4%) across both assessment windows supports the robustness of this pattern. Multimorbidity (2-year window) was present in 285,722 individuals (48.6%) contributing 3,301,572 DALYs (561,565 per 100,000). Table 3 presents the detailed burden breakdown by geriatric syndrome category.

**Table 3:** Geriatric syndromes and associated chronic disease gross DALY burden

Syndrome/Condition	Prevalence *N* (%)	YLL	YLD	Total DALYs	DALY rate per 100,000
Polypharmacy (2-year window)	349,803 (59.5%)	2,193,267	1,842,344	4,035,612	686,408
Polypharmacy (3-year window)	279,842 (47.6%)	1,855,357	1,558,500	3,413,858	580,619
Multimorbidity (2-year window)	285,721 (48.6%)	1,794,333	1,507,239	3,301,572	561,565
PIM Beers 2019 (2-year window)	188,717 (32.1%)	1,221,002	1,025,641	2,246,644	382,118
PIM Thai RDU (2-year window)	24,104 (4.1%)	122,448	102,857	225,305	38,326
Diabetes mellitus (reference)	236,337 (40.2%)	2,342,107	14,169,751	16,511,859	2,910,112
Hypertension (reference)	347,451 (59.1%)	2,192,421	6,138,779	8,331,200	1,417,720
Renal failure (CKD 3b–5) (reference)†	226,931 (38.6%)	1,990,187	1,791,168	3,781,356	643,136

PIM = Potentially Inappropriate Medications. DALY rates per 100,000 population. All estimates are gross population-level burden (unadjusted). †CKD staging corrected to stage 3b–5

### Potentially inappropriate medications

Using 2019 American Geriatrics Society Beers Criteria, PIMs were identified in 188,718 individuals (32.1%), contributing 2,246,644 DALYs (382,118 per 100,000). The most common PIM categories included anticholinergic agents (present in 14.2% of PIM-exposed individuals), sedative/hypnotics including benzodiazepines (11.8%), NSAIDs (9.3%), and sulfonylureas with high hypoglycaemia risk (8.7%). A detailed breakdown by medication class is provided in Supplementary Table S2. Thai-specific RDU criteria identified PIMs in 24,104 individuals (4.1%), contributing 225,305 DALYs (38,326 per 100,000). The discrepancy between criteria (32.1% vs. 4.1%) reflects differences in medication availability, traditional medicine use, and local prescribing patterns in rural Thailand compared to international contexts.

### Disease clustering patterns

Association analysis (Apriori algorithm; minimum support 10%, minimum confidence 60%) identified five major comorbidity clusters. The most prevalent cluster was diabetes–hypertension–renal failure, present in 31.4% of the population (support 0.314, confidence 0.82), followed by hypertension–renal failure–polypharmacy (support 0.28, confidence 0.74). A respiratory–cardiovascular cluster comprising COPD/emphysema, cerebrovascular disease, and mood disorders was identified in 8.3% of individuals (support 0.083, confidence 0.61). A diabetes–polypharmacy–PIM cluster was present in 22.1% (support 0.221, confidence 0.78). A fifth cluster combining diabetes, polypharmacy, PIM exposure, and mood disorders was identified in 12.8% of the population (support 0.128, confidence 0.67), suggesting a complex interplay between metabolic disease, high medication burden, inappropriate prescribing, and psychological comorbidity in this rural older adult population. These clustering patterns represent gross co-occurrence associations and do not imply causal directionality.

## Discussion

### Principal findings

This large-scale population-based study of 587,905 rural Thai older adults accessing outpatient services at secondary hospitals reveals substantial gross disease burden from geriatric syndromes, with polypharmacy affecting nearly 60% and multimorbidity affecting nearly half of the population. Three key findings emerge. First, diabetes mellitus represents the leading disease burden (16.5 million DALYs; 2,910,112 per 100,000) despite lower prevalence than hypertension, highlighting the importance of DALY-based versus prevalence-based priority setting. Second, polypharmacy and multimorbidity contribute gross burdens of 4.0 and 3.3 million DALYs respectively. Third, potentially inappropriate medications affect one-third of the population, with anticholinergics, benzodiazepines, and NSAIDs as the predominant PIM classes, indicating urgent need for medication optimization interventions [41–43].

### Diabetes as leading burden despite lower prevalence

The finding that diabetes (40.2% prevalence) generates twice the gross DALY burden of hypertension (59.1% prevalence) reflects diabetes’ multisystem complications, captured in its high YLD component (85.8% of diabetes DALYs). This aligns with global burden studies [44, 45]. In rural Thailand, this burden is compounded by limited access to specialized diabetes care, inadequate glycemic monitoring, and high rates of diabetic complications [46, 47]. The diabetes–hypertension–renal failure clustering (38.6% renal failure prevalence; CKD stage 3b–5) suggests a cardiometabolic-renal axis driving population burden [48, 49].

### Polypharmacy burden: methodological considerations and clinical implications

The 59.5% polypharmacy prevalence exceeds many international reports (30–50% in community-dwelling older adults) [50, 51]. To minimize overestimation from brand-name switching or generic substitution, polypharmacy was defined at the API level using ATC classification, such that different formulations of the same active ingredient were counted once. Despite this precaution, residual overestimation from herbal medicine co-use and OTC medications recorded in HDC data cannot be excluded and should be considered when interpreting prevalence estimates [52, 53]. Critically, the dominance of YLL (54.3%) in polypharmacy-associated DALYs contrasting sharply with diabetes (YLL 14.2%) and hypertension (YLL 26.3%) suggests that polypharmacy acts more as an accelerator of premature mortality risk than simply a contributor to long-term disability. This pattern is consistent with evidence from systematic reviews demonstrating that polypharmacy independently predicts adverse outcomes including falls, hospitalization, and mortality [54–56]. Recent population-based analyses further confirm increasing inpatient complexity driven by polypharmacy [57, 58].

### Multimorbidity patterns and integrated care

The 48.6% multimorbidity prevalence aligns with global estimates for older populations [59]. The specific clustering patterns particularly diabetes, hypertension, renal disease, and respiratory-cardiovascular combinations have important implications for integrated care delivery in rural settings. Traditional single-disease care models are poorly suited to multimorbid populations, leading to fragmented care, duplicate testing, and medication conflicts [60]. Methodological differences in multimorbidity measurement, including lookback periods and disease counts, substantially influence prevalence estimates and policy interpretation [61, 62].

### Potentially inappropriate medications

The 32.1% PIM prevalence based on Beers 2019 criteria represents a major medication safety concern [63]. Extensive evidence links PIM use to adverse drug reactions, emergency department visits, hospitalization, and increased healthcare costs [64, 65]. In rural Thai contexts, PIM exposure is exacerbated by limited geriatric prescribing expertise, prescribing cascades, continuation of acute-illness medications, and insufficient structured medication review [66, 67]. Anticholinergic and sedative medications, which constitute key PIM categories identified in this study, have been consistently associated with falls and hospitalization. The marked discrepancy between Beers 2019 (32.1%) and Thai RDU (4.1%) criteria underscores the need for context-specific medication appropriateness frameworks [68].

### DALY methodology for geriatric syndromes

Applying the GBD DALY framework to geriatric syndromes such as polypharmacy and multimorbidity is methodologically innovative but requires careful interpretation. In traditional burden of disease analyses, DALYs are estimated for specific diseases or risk factors. Polypharmacy and multimorbidity are conventionally regarded as clinical states or care-complexity indicators, not direct causes of health loss. Standard prevalence-based approaches quantify how many individuals carry these syndromes. They cannot, however, express how much population health loss is associated with them in a manner comparable to disease-level DALY estimates. The present study addresses this gap by applying the GBD DALY framework directly to geriatric syndromes. This generates comparable population health loss metrics, enabling polypharmacy and multimorbidity to be incorporated into evidence-based priority setting alongside traditional diseases. DALYs for geriatric syndromes were estimated using a proxy disability weight derived from the most prevalent associated condition, combined with conservative duration assumptions to yield lower-bound YLD estimates. YLL was subsequently calculated from death records linked to individuals meeting syndrome criteria. These estimates are therefore gross descriptive measures of population health loss associated with rather than caused by these syndromes. Future work should develop syndrome-specific disability weights to refine these estimates [69, 70].

### Strengths and limitations

Strengths include a large population base (*N* = 587,905), comprehensive 25-condition disease classification, validated DALY estimation aligned with GBD 2017 methodology, API-level polypharmacy assessment to minimize brand-substitution overestimation, and a novel focus on geriatric syndromes in rural low- and middle-income settings [71].

**Limitations include**: (1) cross-sectional design precluding causal inference; (2) reliance on outpatient administrative data from secondary hospitals, which likely underestimates the burden in patients managed at tertiary centers or those not accessing formal healthcare this is the most important validity limitation of the study; (3) use of global GBD 2017 disability weights rather than Thai-specific weights; (4) proxy disability weight approach for geriatric syndromes, producing conservative YLD estimates; (5) all DALY estimates are gross (unadjusted) measures they capture total population health loss associated with each condition but cannot isolate the independent contribution of polypharmacy or multimorbidity after controlling for co-occurring diseases; (6) potential overestimation of polypharmacy due to undocumented herbal medicine use; (7) limited generalizability beyond the northeastern Thai context; (8) the selected, healthcare-accessing nature of the study population: the cohort comprises older adults attending outpatient services at 46 secondary hospitals; community-dwelling individuals who do not access formal healthcare are not represented, and the true population-level burden among all rural older adults is likely underestimated; (9) the absence of a comparator group (e.g., younger adults, urban populations, or pre-polypharmacy cohorts), which precludes any inferential comparison all analyses are descriptive and no causal or attributable effect of polypharmacy or multimorbidity on mortality or disability can be inferred from these gross DALY estimates; and (10) administrative data disease misclassification as noted in prior validation studies [72, 73].

### Policy and practice implications

These findings support integrated chronic disease management programs targeting the diabetes–hypertension–renal failure cluster as a priority for rural aging populations. Systematic medication review and deprescribing services should be established as core components of geriatric care. Geriatric pharmacy services delivered via telemedicine represent a feasible and scalable approach to address medication-related burden in rural settings. Implementation of locally validated potentially inappropriate medication (PIM) screening tools is essential to standardize clinical practice [8, 74]. Investment in longitudinal health information systems is critical to support medication reconciliation and enable robust outcome monitoring. Future research should evaluate deprescribing interventions tailored to Thai older adults and develop Thai-specific disability weights for PIM-related adverse outcomes [75, 76].

## Conclusions

This population-based study of 587,905 rural Thai older adults accessing outpatient services at secondary hospitals in northeastern Thailand’s 8th Health Service Region reveals substantial gross disease burden from geriatric syndromes, with polypharmacy and multimorbidity affecting the majority of individuals. Diabetes emerges as the leading disease burden despite lower prevalence than hypertension, highlighting the value of DALY-based priority setting. One-third of the population receives potentially inappropriate medications. These findings apply to older adults within this healthcare-accessing cohort; caution is warranted in extrapolating to the wider rural older adult population, particularly those not accessing formal healthcare services. These gross DALY estimates provide a descriptive population-level picture of health loss associated with each condition and syndrome. They should not be interpreted as independent or causal effect estimates. The high YLL proportion in polypharmacy-associated DALYs warrants particular attention. These findings underscore the necessity of integrated, geriatric-focused care models and pharmaceutical services adapted to rural aging populations in low- and middle-income countries.

## Supplementary Information

Below is the link to the electronic supplementary material.

Supplementary Material 1

## Data Availability

De-identified data are available upon reasonable request to qualified researchers, subject to approval by data custodians and appropriate data sharing agreements.
