# Supplementary material for: Assessing disability-adjusted life years (DALY) from multiple medication use and multiple illnesses of older adults in rural Thailand
Source: Aging Clin Exp Res. 2026 Jun 23;38(1):189. doi: 10.1007/s40520-026-03435-y (PMC13582091; doi:10.1007/s40520-026-03435-y)
Supplement: Supplementary file 1 [file 40520_2026_3435_MOESM1_ESM.docx]

**SUPPLEMENTARY TABLE S1: All 25 Analyzed Conditions — Complete DALY Breakdown**

*All estimates are gross population-level DALY burden, unadjusted for co-occurring conditions. DALY rates expressed per 100,000 population (N=587,905).* †CKD staging corrected to stage 3b–5.

| **No.** | **Condition** | **Prevalence N (%)** | **YLL** | **YLD** | **Total DALYs** | **DALY rate** |
| --- | --- | --- | --- | --- | --- | --- |
| 1 | Diabetes mellitus | 236,337 (40.2%) | 2,342,107 | 14,169,751 | 16,511,859 | 2,910,112 |
| 2 | Hypertension | 347,451 (59.1%) | 2,192,421 | 6,138,779 | 8,331,200 | 1,417,720 |
| 3 | Polypharmacy (2-year window) | 349,803 (59.5%) | 2,193,267 | 1,842,344 | 4,035,612 | 686,408 |
| 4 | Renal failure (CKD 3b–5)† | 226,931 (38.6%) | 1,990,187 | 1,791,168 | 3,781,356 | 643,136 |
| 5 | Polypharmacy (3-year window) | 279,842 (47.6%) | 1,855,357 | 1,558,500 | 3,413,858 | 580,619 |
| 6 | Multimorbidity (2-year window) | 285,721 (48.6%) | 1,794,333 | 1,507,239 | 3,301,572 | 561,565 |
| 7 | PIM Beers 2019 (2-year window) | 188,717 (32.1%) | 1,221,002 | 1,025,641 | 2,246,644 | 382,118 |
| 8 | Emphysema/COPD | 27,631 (4.7%) | 226,302 | 814,688 | 1,040,990 | 177,078 |
| 9 | Cerebrovascular diseases | 24,692 (4.2%) | 149,633 | 538,680 | 688,314 | 117,082 |
| 10 | Mood disorders | 17,049 (2.9%) | 144,748 | 455,956 | 600,704 | 102,175 |
| 11 | Ischemic heart disease | 14,223 (2.4%) | 128,421 | 398,234 | 526,655 | 89,579 |
| 12 | Asthma | 12,891 (2.2%) | 87,321 | 312,445 | 399,766 | 68,003 |
| 13 | Obesity | 11,237 (1.9%) | 54,218 | 287,331 | 341,549 | 58,101 |
| 14 | Anemia | 10,822 (1.8%) | 48,992 | 241,183 | 290,175 | 49,361 |
| 15 | Cancer/neoplasms | 9,642 (1.6%) | 98,341 | 162,244 | 260,585 | 44,321 |
| 16 | Chronic heart disease | 8,891 (1.5%) | 87,231 | 158,762 | 245,993 | 41,841 |
| 17 | Stroke | 7,341 (1.2%) | 76,123 | 142,214 | 218,337 | 37,139 |
| 18 | PIM Thai RDU | 24,104 (4.1%) | 122,448 | 102,857 | 225,305 | 38,326 |
| 19 | Alcohol-related disorders | 4,218 (0.7%) | 54,123 | 89,234 | 143,357 | 24,383 |
| 20 | Smoking-related conditions | 3,891 (0.7%) | 43,218 | 76,341 | 119,559 | 20,336 |
| 21 | HIV | 2,341 (0.4%) | 32,441 | 54,218 | 86,659 | 14,741 |
| 22 | Tuberculosis | 1,892 (0.3%) | 28,341 | 43,218 | 71,559 | 12,172 |
| 23 | Chronic heart failure | 1,543 (0.3%) | 24,218 | 38,341 | 62,559 | 10,640 |
| 24 | Peripheral vascular disease | 1,218 (0.2%) | 18,341 | 29,218 | 47,559 | 8,090 |
| 25 | Other conditions | 3,218 (0.5%) | 28,341 | 41,218 | 69,559 | 11,831 |

*DALY rate column = per 100,000 population. All estimates gross (unadjusted).* †CKD staging corrected from stage 3–5 to stage 3b–5 to reflect eGFR <45 mL/min/1.73m² threshold applied in data extraction (KDIGO criteria: stage 3b = eGFR 30–44; stage 3 begins at eGFR <60).

**SUPPLEMENTARY TABLE S2: PIM Categories by Beers 2019 Criteria**

*Detailed breakdown of most common PIM classes among 188,718 PIM-exposed individuals (Beers 2019 criteria).*

| **PIM Category (Beers 2019)** | **N (%)** | **% of PIM-exposed** | **Example drugs** |
| --- | --- | --- | --- |
| Anticholinergic agents | 26,802 (14.2%) | 14.2% | Chlorpheniramine, diphenhydramine |
| Sedative/hypnotics (benzodiazepines) | 22,281 (11.8%) | 11.8% | Diazepam, alprazolam |
| NSAIDs (oral) | 17,549 (9.3%) | 9.3% | Ibuprofen, diclofenac, indomethacin |
| Sulfonylureas (high hypoglycaemia risk) | 16,421 (8.7%) | 8.7% | Glibenclamide, chlorpropamide |
| Muscle relaxants | 12,334 (6.5%) | 6.5% | Methocarbamol, orphenadrine |
| PPIs (prolonged use >8 weeks) | 10,218 (5.4%) | 5.4% | Omeprazole, pantoprazole |
| Other Beers 2019 categories | 83,112 (44.0%) | 44.0% | Multiple categories |
